# Commonalities between the Atacama Desert and Antarctica rhizosphere microbial communities

**DOI:** 10.3389/fmicb.2023.1197399

**Published:** 2023-07-19

**Authors:** María José Contreras, Karla Leal, Pablo Bruna, Kattia Nuñez-Montero, Olman Goméz-Espinoza, Andrés Santos, León Bravo, Bernardita Valenzuela, Francisco Solis, Giovanni Gahona, Mayra Cayo, M. Alejandro Dinamarca, Claudia Ibacache-Quiroga, Pedro Zamorano, Leticia Barrientos

**Affiliations:** ^1^Centro de Excelencia en Medicina Traslacional, Facultad de Medicina, Universidad de La Frontera, Temuco, Chile; ^2^Instituto de Ciencias Aplicadas, Facultad de Ingeniería, Universidad Autónoma de Chile, Temuco, Chile; ^3^Biotechnology Research Center, Instituto Tecnológico de Costa Rica, Cártago, Costa Rica; ^4^Department of Agricultural Sciences and Natural Resources, Faculty of Agricultural Sciences and Environment, Universidad de La Frontera, Temuco, Chile; ^5^Universitat Autònoma de Barcelona, Departament de Genètica i de Microbiologia, Institut Biotecnologia i de Biomedicina, Cerdanyola del Vallès, Barcelona, Spain; ^6^Laboratorio de Microorganismos Extremófilos, Instituto Antofagasta, Universidad de Antofagasta, Antofagasta, Chile; ^7^Escuela de Nutrición y Dietética, Facultad de Farmacia, Universidad de Valparaíso, Valparaíso, Chile; ^8^Centro de Micro-Bioinnovación, Universidad de Valparaíso, Valparaíso, Chile; ^9^Departamento Biomédico, Facultad de Ciencias de la Salud, Universidad de Antofagasta, Antofagasta, Chile

**Keywords:** rhizosphere, metabarcoding, Atacama Desert, the Antarctica, plant associated-bacteria

## Abstract

Plant-microbiota interactions have significant effects on plant growth, health, and productivity. Rhizosphere microorganisms are involved in processes that promote physiological responses to biotic and abiotic stresses in plants. In recent years, the interest in microorganisms to improve plant productivity has increased, mainly aiming to find promising strains to overcome the impact of climate change on crops. In this work, we hypothesize that given the desertic environment of the Antarctic and the Atacama Desert, different plant species inhabiting these areas might share microbial taxa with functions associated with desiccation and drought stress tolerance. Therefore, in this study, we described and compared the composition of the rhizobacterial community associated with *Deschampsia antarctica* (Da), *Colobanthus quitensis* (Cq) from Antarctic territories, and *Croton chilensis* (Cc), *Eulychnia iquiquensis* (Ei) and *Nicotiana solanifolia* (Ns) from coastal Atacama Desert environments by using 16S rRNA amplicon sequencing. In addition, we evaluated the putative functions of that rhizobacterial community that are likely involved in nutrient acquisition and stress tolerance of these plants. Even though each plant microbial rhizosphere presents a unique taxonomic pattern of 3,019 different sequences, the distribution at the genus level showed a core microbiome with a higher abundance of *Haliangium, Bryobacter, Bacillus*, MND1 from the *Nitrosomonadaceae* family, and unclassified taxa from *Gemmatiamonadaceae* and *Chitinophagaceae* families in the rhizosphere of all samples analyzed (781 unique sequences). In addition, species *Gemmatirosa kalamazoonesis* and *Solibacter usitatus* were shared by the core microbiome of both Antarctic and Desert plants. All the taxa mentioned above had been previously associated with beneficial effects in plants. Also, this microbial core composition converged with the functional prediction related to survival under harsh conditions, including chemoheterotrophy, ureolysis, phototrophy, nitrogen fixation, and chitinolysis. Therefore, this study provides relevant information for the exploration of rhizospheric microorganisms from plants in extreme conditions of the Atacama Desert and Antarctic as promising plant growth-promoting rhizobacteria.

## 1. Introduction

Plants host a diverse microbial community. Their interactions are essential for thriving in any environment. These microbial communities are mainly composed of bacteria and fungi necessary for plant nutrition and resistance to biotic and abiotic stresses (Fujiwara et al., 2023). Therefore, the plant’s fitness results from the plant and its symbiotic interaction with its accompanying microbiota, together known as Holobiont (Vandenkoornhuyse et al., 2015). Plant-microbiota interactions include mutualism, commensalism, and antagonism relations. These relationships have significant effects on plant growth, health, and productivity. Commonly, microorganisms benefit from nutrients secreted by the plants in the surrounding environments, allowing community growth and diversification. On the other hand, the microorganisms collaborate with plants for water and nutrient absorption process. Among other activities that promote plant growth, plant-associated microbes also prevent or compete with plant pathogens and some of them can secrete molecules such as exopolysaccharides, which contribute to water retention (Seth et al., 2023).

Particularly, rhizosphere microorganisms are involved in processes that promote physiological responses to biotic and abiotic stresses in plants, such as nitrogen fixation (Chandra et al., 2021). Plant-microbe associations have been described to trigger plant processes and morphology modifications, including increased tolerance to stress conditions (Qiao et al., 2017). Hence, rhizosphere microorganisms have been proposed as drivers of plant tolerance and resistance to pests, pathogens, drought, salinity, and pH imbalances, sometimes providing increased resistance to these conditions (Goswami and Deka, 2020). In this sense, the close relationship between the plant and the rhizosphere microorganisms through active communication signaling pathways could influence plant success in the surrounding environment. Therefore, the study of the holobiont has regained relevance; microorganisms such as endophytes, symbionts, pathogens, and plant growth-promoting rhizobacteria (PGPRs), are increasingly featured in the literature.

‘In recent years, the study of plants growing in these extreme environments and their holobiont have increased (Alsharif et al., 2020). About 33% of the Earth’s surface is desert. In these arid regions plant must overcome multiple adverse environmental conditions to survive. Extreme environments perturb interactions between species and force species to adapt, migrate, be replaced by others, or go extinct. Despite the harsh conditions, many microorganisms can proliferate under extreme temperatures, salinity, pH, and pressure; these microorganisms are called extremophiles (Madigan, 2000). Also, certain species of plants can inhabit some poly-extreme environments, and the role of their microbiomes might be linked to their resistance, which has gained attention in multiple recent studies (Dikilitas et al., 2021). For instance, bacteria associated with plants can alleviate plant stress through various mechanisms. They can produce phytohormones, such as auxins, cytokinins, and gibberellins, which promote plant growth (Muhammad et al., 2022). Additionally, these bacteria enhance the accumulation of solutes like trehalose, proline, and glycine betaine, which function as osmolytes and help maintain turgor pressure within plant cells during drought stress. Moreover, they also regulate genes involved in stress responses, photosynthesis, and other metabolic pathways (Morcillo and Manzanera, 2021; Muhammad et al., 2022).

One of the great arid regions on Earth is the Atacama Desert, which stretches 1,000 km from 20°S to 30°S on the Pacific coast of South America, between the Andean Mountain Range and the coast of Chile. This is characterized as an extremely arid environment due to the strong presence of high-pressure centers in the atmosphere on the South Pacific coast of America, showing a constant temperature inversion due to the cold Humboldt current in the ocean. Geological and mineralogical evidence suggests that the conditions of extreme aridity have persisted for more than 10 million years (McKay et al., 2003). The coastal desert also presents areas with low rainfall and a strong influence of coastal fog, locally called “camanchaca.” This is because the atmospheric boundary layer along the arid coast of northern Chile is crowned by a pronounced inversion temperature at about 1,000 m.s.n.m., which separates the humid marine air and the much drier air from the desert, generating humidity inputs for a few hours, while local radiation generates constant drought fluctuations (Schulz et al., 2012). This is the case in the Paposo area, north of the city of Taltal, classified as dry coastal. Despite the harsh environment, a unique vegetation of endemic species subsists on the coastal cliffs, with more than 250 species of vascular plants (Rundel et al., 2007). The biodiversity of organisms that inhabit this area is adapted to extreme conditions related to aridity, height, mineralization, and temperature variations (Navarro-González et al., 2003; Okoro et al., 2009).

On the other hand, cold deserts, characteristic of Antarctica, also present harsh environmental conditions like low temperatures, low water content, low nutrient availability, and saline soils (Convey et al., 2008; Gallardo-Cerda et al., 2018). In such conditions, only two native vascular plants, *Colobanthus quitensis* and *Deschampsia antarctica,* inhabit the area (Alberdi et al., 2002). In the last ten years, the interest in microorganisms associated with these vascular plants has dramatically increased, finding promising bacterial and fungal strains with a broad spectrum of applications (Parnikoza et al., 2011; Fardella et al., 2014; Molina-Montenegro et al., 2016; Santiago et al., 2017).

In this context, we hypothesize that given the desertic environment of both regions (i.e., the Antarctic and the Atacama Desert), different plant species inhabiting these areas might share microbial taxa associated with plant survival under stress conditions present in both ecosystems desiccation, drought, and extreme temperatures. Therefore, in this study, we described and compared the composition of the rhizobacterial community associated with *Deschampsia antarctica* (Da), *Colobanthus quitensis* (Cq) from Antarctic territories, and *Croton chilensis* (Cc), *Eulychnia iquiquensis* (Ei) and *Nicotiana solanifolia* (Ns) from Atacama Desert by using 16S rRNA gene amplicon sequencing in order to identify the bacterial community commonalities. In addition, we also evaluated the putative functions of that rhizobacterial community that are likely involved in nutrient acquisition and stress tolerance.

## 2. Materials and methods

### 2.1. Sampling sites

**Figure 1:**
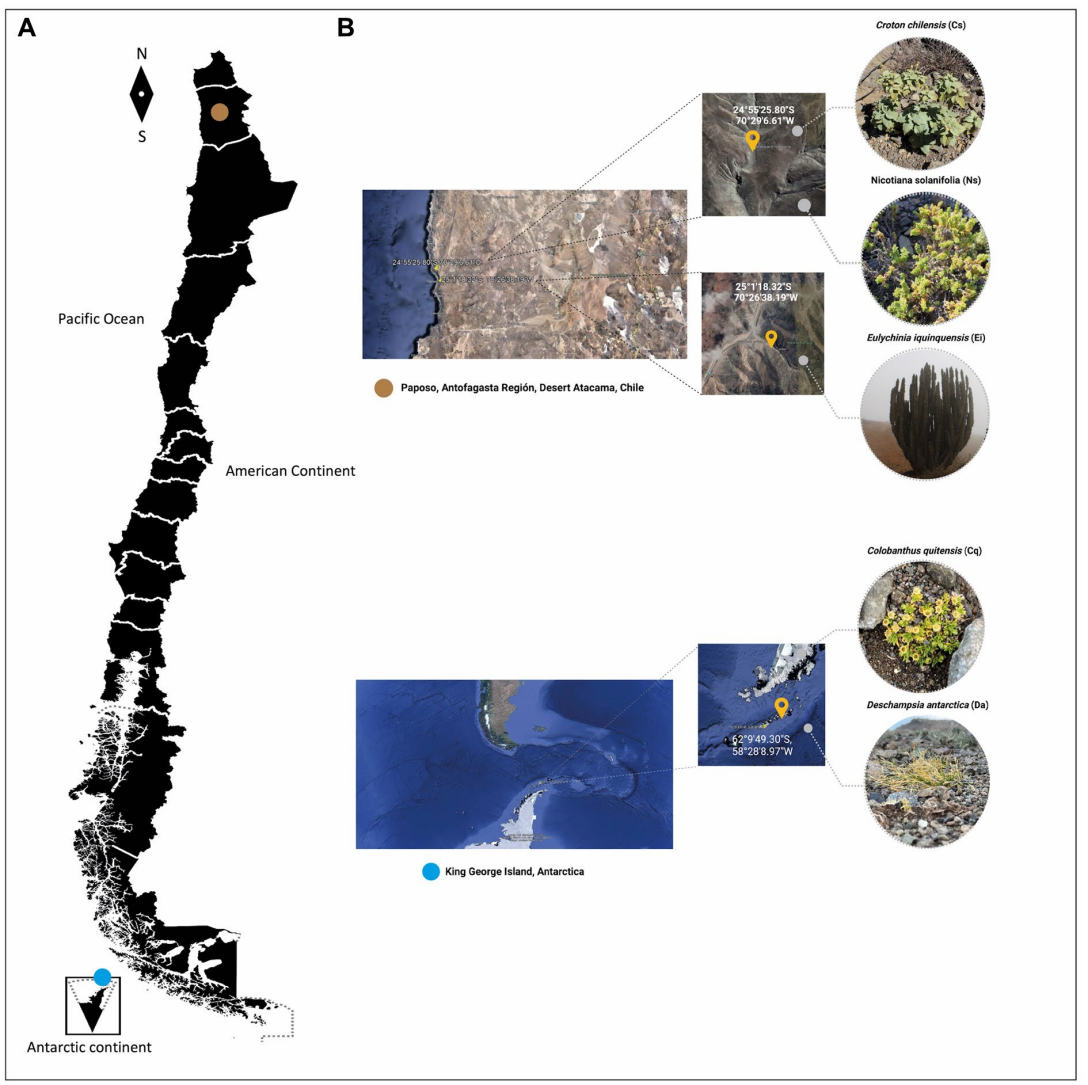
**(A)** Figure representing the map of Chile and sampling sites, brown circle (Atacama Desert), blue circle (Antarctica). **(B)** Croton chilensis (CC), Eulychnia iquiquensis (Ei), and Nicotiana solanifolia (Ns) from the Atama Desert and Deschampsia Antarctica (Da) and Colobantus quitensis (Cq) from Antarctica.

Since only two vascular plants grow in Antarctica (*Colobanthus quitensis* and *Deschampsia Antarctica*), both were selected to be included in this study. Also, *Croton chilensis, Eulychnia iquiquensis* and *Nicotiana solanifolia*, were selected as model of vascular plants inhabiting Atacama Desert. Rhizosphere soil samples were collected, in triplicate, between February and May 2022 from each of the species mentioned above in the Atacama Desert and Antarctic regions. The sampling location and characteristics of each site and sample are specified in Figure 1. Three quadrants (5 × 5 m per quadrant) were established in each site as three rhizosphere cores. In each quadrant, three plants with their rhizosphere were randomly collected and mixed to form a composite soil rhizosphere sample. During sampling, the rhizosphere soil from each plant was carefully removed by excavating the plants’ roots zone to a depth of 0–20 cm using a sterilized spade to collect the soil at about 2 mm from the roots and placing the composite soil samples into sterile polyethylene sterile bags. Soil samples were refrigerated at 4°C, immediately transported to the laboratory, and stored at −80\u00B0C until used for DNA extraction.

### 2.2. DNA extraction from rhizosphere samples

Total environmental DNA was extracted from rhizosphere samples. 0.5 mg of collected soil samples were processed with Dneasy® PowerSoil DNA isolation kits (QIAGEN, United States) using the bead beating protocol according to the manufacturer’s instructions. The quality and quantity of the DNA extracts were measured using a QuantiFluor dsDNA system (Promega, United States), with the optimal concentration of <10 ng/uL per sample. Subsequently, genomic DNA integrity was assessed with 1% agarose gel.

### 2.3. The library preparation and nanopore sequencing

The 16S sequencing library was prepared with the 16S rapid amplicon barcoding kit SQK-RAB204, following the manufacturer’s recommended procedures (Oxford Nanopore Technologies-ONT, UK). The complete 16S rRNA gene was amplified using 10 ng/uL of total DNA from all samples. LongAmp® Taq 2× (New England Biolabs, United States) was used to perform PCR using the primer pair (27F and 1492R) provided by ONT. Amplifications were carried out on a MultiGene™ OptiMax Thermal Cycler (Labnet, United States), using the program: 1 min denaturation at 95°C, 35 cycles (95°C-20 s, 55°C- 30 s, 65°C – 1 min), and a final extension step of 5 min at 65°C. The 16S rRNA gene amplicons were quantified using the QuantiFluor dsDNA system (Promega, USA), pooled in equal amounts (100 fmoles) per sample, and the library was further processed as described by the manufacturers. Subsequently, the library was incubated with Library Loading Beads (Oxford Nanopore Technologies-ONT, UK), and the mixture was added to the MinION flow cell (version R9.4) (Oxford Nanopore Technologies-ONT, UK). Nanopore libraries were sequenced using a MinION Mk1C with the recommended specifications for nanopore sequencing. All samples were sequenced on R9.4 flow cells and the recommended scripts in MinKNOW to generate fast5 files with live basecalling enabled.

### 2.4. Data analysis

16S rRNA gene amplicons were processed and analyzed following previously recommended tools for metabarcoding using third-generation sequencing (Santos et al., 2020). Adapter sequences were trimmed from the reads using Porechop,^1^ and a filter was performed using Nanofilt v2.8.0 with length over 1,000 bp, max length on 1900 bp and quality >9 (De Coster et al., 2018). Direct taxonomic assignment of the sequences was performed using Centrifuge v1.0.4 against the SILVA 138 SSU database for all the samples and their respective replicates, using default parameters (Kim et al., 2016). Species-level analyses were conducted with Emu (Curry et al., 2022) using the –keep-counts option and the NCBI 16S RefSeq database. Species abundance tables were obtained using a custom script. Finally, plots and diversity analyses were carried out using MicrobiomeAnalyst. Subsequently, the data were further processed with Pavian package v1.2.0 for data exploration and visualization (Breitwieser and Salzberg, 2020). Data of the raw 16S rRNA gene sequences are available at the Genbank repository under the Bioproject accession PRJNA972307.

Functional prediction analyses were performed employing sequences using FAPROTAX v1.2.6 (Louca et al., 2016). That is a manually constructed database that maps prokaryotic taxa (e.g., genera or species) to metabolic or other ecologically relevant functions based on the literature on cultured representatives. It includes 7,600 functional annotations covering over 4,600 taxa and over 80 functions such as nitrate respiration, methanogenesis, fermentation, or plant pathogenesis. Functions represented in FAPROTAX focus on marine and lake biogeochemistry, particularly sulfur, nitrogen, hydrogen, and carbon cycling, although other functions are also included (Louca et al., 2016). It should be noted that predictions are based on cultivable evidence, so predictions of unculture taxa are based on the closest genus and should be validated with further study. FAPROTAX script (collapse_table.py) is available online,^2^ and it was used for converting prokaryotic taxa abundance profiles (species table) into putative functional group abundance profiles, based on the taxa identified at the species level.

### 2.5. Statistical analyses

Statistical analysis of sequences with the taxonomic assignment was visualized using MicrobiomeAnalyst (Dhariwal et al., 2017; Xu X. et al., 2020; Xu R. et al., 2020). To perform alpha-diversity analyses, each sample was normalized using maker data profiling (MDP) to generate Chao1, Shannon, Simpson, and Fisher indices (Dhariwal et al., 2017). Data were confirmed for normality using the Shapiro–Wilk test (*p* > 0.05). A one-way ANOVA test of variance (*p* < 0.05) was performed to compare the alpha diversity among compartments. All statistical analyses were done using GraphPad Prism 9 (GraphPad Software, La Jolla, CA, United States). Differences in community composition and Principal Coordinates Analysis (PcoA) were calculated using the Bray-Curtis Index with rarefied communities using MicrobiomeAnalyst internal scripts. Permanova (*p* < 0.05) was performed to compare PcoA. A Venn diagram of the sequences shared between each sample was conducted using Bioinformatics & Evolutionary Genomics (bioinformatics.psb.ugent.be/webtools/Venn).

## 3. Results

A total of 3,019 16S rRNA sequences were observed, which were allocated in Da (1379), Cq (1336), Cc (1711), Ei (1437), and Ns (1495). Taxonomic assignment of the sequences of all plants revealed that the main taxa belonged to *Proteobacteria, Bacteroidota, Acidobacteriota*, and *Planctomycetota* (Supplementary Figure S1A). However, there were differences between the samples from Antarctica and the desert regarding the most abundant taxa in the rhizosphere of each plant. Preceded by *Proteobacteria*, the Antarctic samples had more representation of *Bacteroidota, Acidobacteriota,* and *Myxococcota*. However, in the desert rhizosphere samples, *Planctomycetota, Actinobacteriota,* and *Firmicutes* were predominant (Supplementary Figure S1A).

The distribution percentage of bacterial taxa were also determined at the genus level, showing a higher presence of *Haliangium*, *Bryobacter*, *Bacillus*, and unclassified taxa from the *Isosphaeraceae*, *Gemmatiamonadaceae,* and *Chitinophagacea*e families among the different samples (Supplementary Figure S2B). The rhizosphere of Antarctic plants, Da and Cq, showed a higher presence of *Haliangium, Bryobacter*, and unclassified genera from the *Chitinophageceae* and *Puia* families, compared to the desert samples. In contrast, the desert rhizosphere from Cc, Ei, and Ns showed a higher relative abundance of the genus *Bacillus*, *MND1,* and unclassified taxa from *Gemmatiamonadaceae* and *Isosphaeraceae* families.

**Figure 2:**
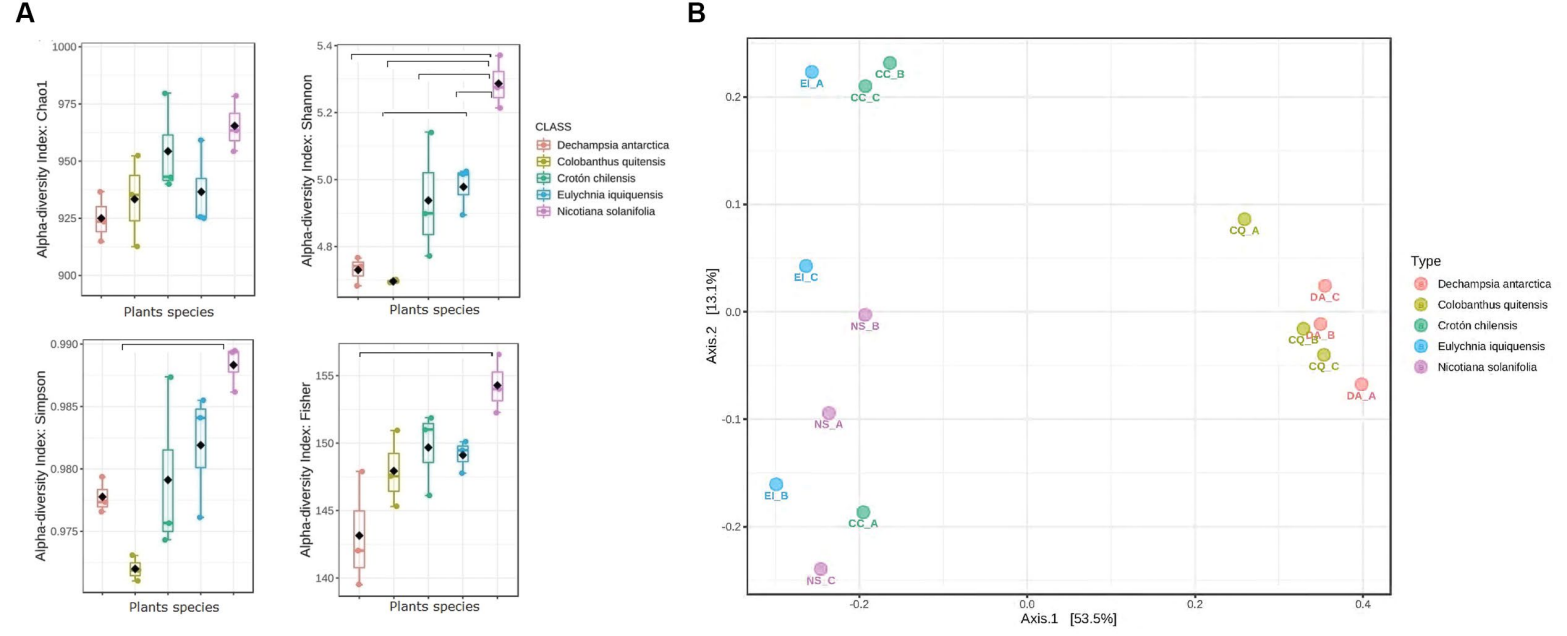
Bacterial communities’ diversity analysis among in the rhizosphere of Antarctic and Atacama Desert plants and functional prediction. **(A)** Boxplot showing alpha diversity analyses (Chao1, Shannon, Simpson, and Fisher) in rhizosphere of Da, Cq, Cc, Ei, and Ni samples. The horizontal bars within the boxes represent the median and are considered as a value of *p* >0.05. **(B)** PCoA of bacterial communities based on the Bray-Curtis Index, as affected by plant species. (Permanova, value of *p* <0.001).

The alpha diversity indices evidenced no statistical difference regarding the Chao1 index (Figure 2A). However, differences were observed on Shannon and Simpson indices, with the highest values found in the desert rhizosphere samples (ANOVA value of *p* = 0.00017 and value of *p* = 0.008, respectively). These results confirmed a greater diversity in desert taxa than in Antarctic samples. Also, diversity based on Fisher was significantly higher for the rhizosphere of Ns samples from the desert.

In addition, a PCoA analysis was performed to determine the taxonomic variation according to plant species across the samples (Figure 2B). The plots revealed a separation of the rhizosphere of plants according to their origin from the Atacama Desert (Cc, Ei, and Ns) or the Antarctic (Da and Cq). However, PC1 and PC2 explained only 53.5 and 13.1% of the total variance, respectively, implying the existence of other factors that could affect bacterial communities in the rhizosphere of Antarctic and Atacama Desert plants, which is expected since we worked with different plant species.

**Figure 3:**
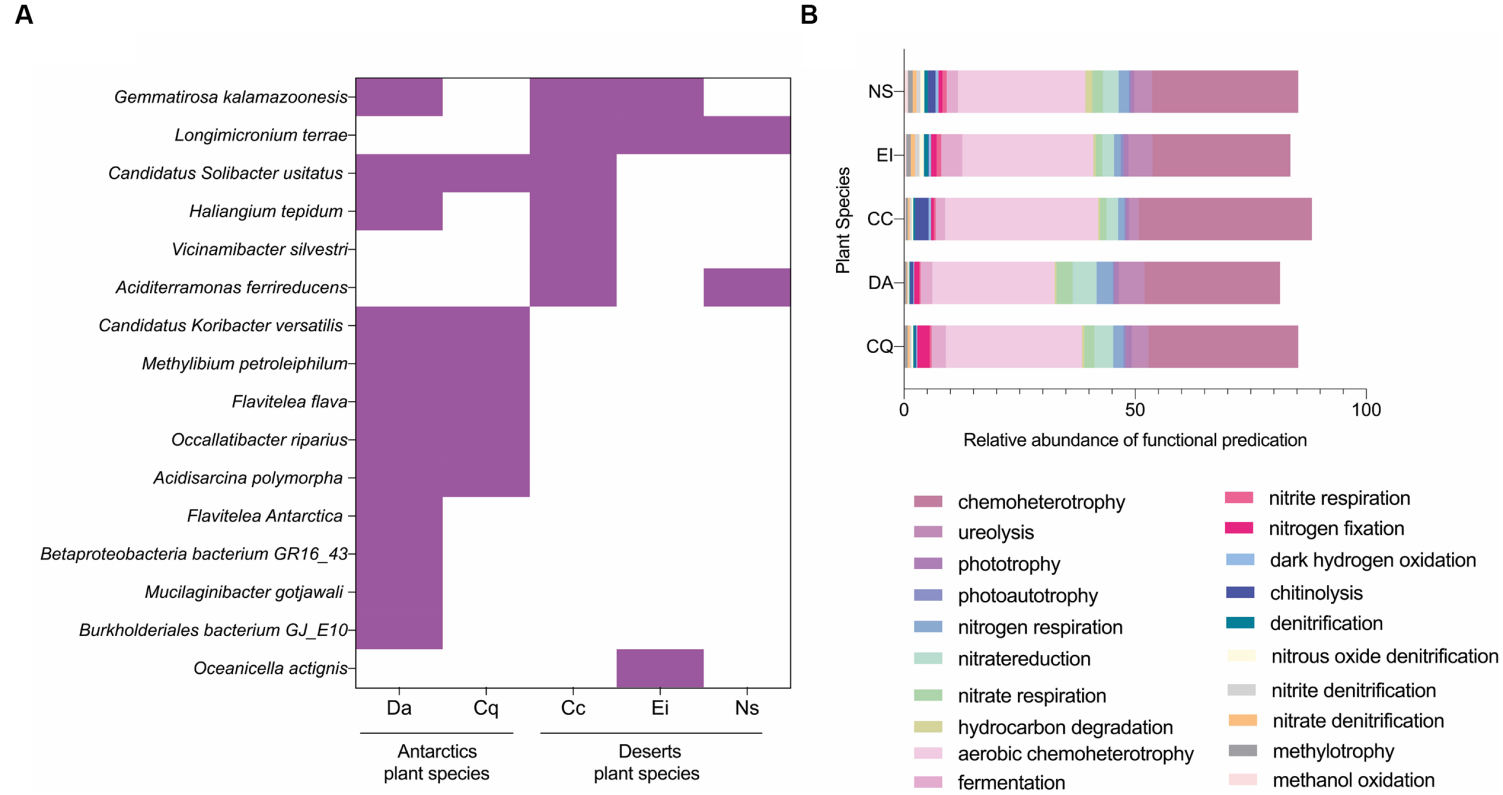
Core rhizosphere microbiome and its functional prediction. **(A)** Heatmap matrix of core bacterial species found across the five plants under study. **(B)** Most represented functions predicted from the 781 shared sequences across the 5 plant rhizospheres (commonalities) based on functional prediction analysis performed with FAPROTAX v1.2.6. *Deschampsia Antarctica (Da), Colobantus quitensis (Cq), Croton chilensis (Cc), Eulychnia iquiquensis (Ei), Nicotiana solanifolia (Ns)*.

Next, the core microbiome was analyzed at the species level, defined as the common bacterial species shared by all the individuals for each plant species. The total of core bacterial species ranged from two to 12 depending on the plant species (Supplementary Figure S2). Six shared species were found in the core microbiomes of the Antarctic samples: *Candidatus Solibacter usitatus, Candidatus Koribacter versitalis, Flavitalea flava, Methylibium petroleiphilum, Occallatibacter riparius,* and *Acidisarcina polymorph*. In comparison, *Longimicrobium terrae* was shared in the core microbiome of the Atacama Desert plants. Also, only two species (*Gemmatirosa kalamazoonesis* and *Candidatus Solibacter usitatus*) were found to be shared by a maximum of three plant species from both Antarctic and Desert origin (Figure 3A).

A total of 781 16S rRNA gene sequences were shared between the rhizosphere bacterial communities of Antarctic and Atacama Desert plants, equivalent to approximately 26% of the total sequences. In addition, despite the commonalities between samples, each plant species showed a distinct fingerprint. Within the Antarctic group, Da had 80, and Cq had 72 unique taxa. In the case of the desert samples, CC had 155, Ei had 109, and Ni had 168 unique taxa, not repeated with the other plant species (Supplementary Figure S1E). The composition of shared taxa was determined to identify common unique sequences in the rhizosphere of five desertic species. The main phylum shared among all rhizospheres and in similar abundance were *Proteobacteria* and *Bacteriodota*, followed in different proportions by *Acidobacteriota*, *Planctomycetota*, *Myxococcota,* and *Actinobacteriota* (Supplementary Figure S1C). Meanwhile, the common genera and most abundant for all rhizospheres were *Haliangium, Bacillus, Bryobacter*, MND1 from the *Nitrosomonadaceae* family, and unclassified taxa from *Gemmatiamonadaceae* and *Chitinophagaceae* families (Supplementary Figure S1D).

Predictions of functional potential showed a predominance of chemoheterotrophy, ureolysis, phototrophy, nitrogen respiration, and nitrate reduction. Slight differences in functions were observed concerning the origin of the sample. In the Atacama Desert rhizosphere, predicted functions of chitinolysis and aerobic chemoheterotrophy were more represented than in the Antarctic plants; meanwhile, nitrate reduction, nitrate respiration, and nitrogen fixation were predicted mostly for the Antarctic rhizosphere communities (Supplementary Figure S1F). No major differences were observed when the functions were predicted only for the bacterial commonalities (781 shared unique sequences, Figure 3B).

## 4. Discussion

Our comprehension of microbial communities has improved with the advances in sequencing technologies. Particularly, third-generation sequencing has facilitated the obtention of longer reads, providing potentially higher accuracy and a more extensive understanding of the microbial realm. These advances have also unlocked new avenues for research and exploration, with the potential to unveil novel insights into the functions of microbes in diverse ecosystems, including extreme environments such as the Antarctic and the Atacama Desert (Singh and Shankar, 2021). Consequently, our study aimed to characterize 16S rRNA gene sequences and compare the taxonomic and functional diversity of the microbial communities of two Antarctic vascular species, Da and Cq, and three Atacama Desert vascular species, Cc, Ei, and Ni, which are of great interest because of their high tolerance to extreme conditions such as drought, salinity, UV radiation, and extreme temperature.

The results obtained in our study revealed a large relative abundance of *Proteobacteria, Bacteroidetes, Acidobacteria*, and *Planctomycetota* in both ecosystems, the Antarctic, and the Atacama Desert. Some scientific research has investigated bacterial diversity in the rhizosphere soil of Antarctic vascular plants using 16S rRNA gene sequencing techniques. Molina-Montenegro et al. (2019) performed a study comparing the rhizosphere of Antarctic plants Cq and Da using an Illumina sequencing-based approach. Their analysis revealed a wide diversity of microbial communities at the phylum level, with the most abundant being *Proteobacteria* (34.1%), *Actinobacteria* (23.7%), and *Bacteroidetes* (16.4%). Similarly, our results showed that the most abundant phyla in Da and Cq were *Proteobacteria* and *Bacteroidetes*. Several studies agree that the most abundant taxa in Antarctic soil samples are *Acidobacteria, Actinobacteria*, *Proteobacteria* (most commonly *Alpha*– and *Gammaproteobacteria*), *Bacteroidetes*, and *Firmicutes* (Ganzert et al., 2011; Lee et al., 2012). However, differences in the abundance of these phyla were observed between samples. This result could be mainly due to the microenvironment of the site and the geographical location of the samples studied in Antarctica, as previously reported in similar studies (Doytchinov and Dimov, 2022).

In contrast, to our knowledge, no previous studies about the rhizosphere of Atacama Desert plants, such as Cc, Ni, and Ei species, use culture-independent techniques. However, reports have shown the rhizosphere bacterial composition of other vascular species living in different locations in the Atacama Desert. Fernández-Gómez et al. (2019) compared the rhizosphere of four native plant species: *Calamagrostis crispa, Nassella nardoides, Jarava frigida* and *Pycnophyllum bryoides*, located in the Atacama Salt Flat and adjacent Andes. Their results revealed that the phylum *Proteobacteria* dominated the rhizosphere, mainly comprising alpha (28%) and *Actinobacteria* (mainly *Blastocatellia*). In addition, a recent study was carried out in the rhizosphere of the plant *Parastrephia quadrangularis*, obtained from the slopes of the volcanoes Guallatiri, Isluga, and Lascar, located in the Atacama Desert, reporting that the most abundant phyla were *Actinobacteria, Proteobacteria, Acidobacteria* and *Bacteroidetes* (Zhang et al., 2021). Both studies agree with our results, showing a higher abundance of the phyla *Proteobacteria* and *Actinobacteria*. Despite similarity at phylum level, differences in bacterial diversity were demonstrated at lower taxonomic ranks. Shannon, Simpson, and Fisher diversity indices showed higher diversity in the rhizosphere of plants from the Atacama Desert. Although the Chao1 index does not show significant differences, this outcome is anticipated as the index primarily assesses species richness, estimating the total number of unique species in a given sample. In contrast, the Shannon, Simpson, and Fisher indices consider species richness and evenness, which is the relative abundance of various species within the sample, offering a greater sensitivity as diversity metrics. These results agree with other studies where Antarctic diversity was reported to be lower than other soil systems, probably as the result of selection imposed by the generally cold, dry, and oligotrophic nature of the Antarctic (Chown et al., 2015; Okie et al., 2015; Lee et al., 2018).

A total of 781 sequences were shared in the rhizosphere of both Antarctic and Atacama Desert plants (~26% of total unique sequences). No other studies have identified unique shared sequences using the complete 16S rRNA gene among the rhizosphere of different plant species. However, similar approaches using partial 16S rRNA sequencing have been employed to compare the microbial communities in the rhizosphere of plants. For example, one study compared the microbial communities between rusty and healthy ginseng roots, observing significant changes in the healthy condition. They found that only 20.5% of amplicon sequence variants (ASVs) were shared between the two conditions (Bian et al., 2020). Another study compared the rhizosphere microbiome of six plant species from two orders and three families of wild plants grown in the same field. Approximately 39% of the plant taxonomic operational units (OTUs) were found to be part of the common rhizosphere microbiome (Lei et al., 2019). Furthermore, other authors examined the rhizosphere of four desert halophytes and reported high variability of phylotypes (at the genus level). They discovered that these plant species shared less than 6% of the identified phylotypes (Mukhtar et al., 2021).

Given the high variability of the rhizosphere microbiome, influenced by factors such as plant species, site characteristics, and environmental conditions, the common taxa reported here might play a crucial role in the adaptation of extreme-tolerant plants to their specific environments.

Among the commonalities found in this study, the genera *Haliangium, Bacillus, Bryobacter,* MND1, and members of *Gemmatiamonadaceae* and *Chitinophagaceae* families appeared relevant to all five plant species. Some of those taxonomic groups have already been reported to benefit plant growth under complex conditions. A recent study shows an increase in *Gemmatimonadetes* and *Firmicutes* as a response to the herbicide application and an increase in the genus *Bryobacter* responsible for metabolizing the chemical compounds (Pertile et al., 2021). *Bryobacter* spp. has also been associated with healthy plants and is a primary driver of bacterial structure (Wang R. et al., 2022). A new study suggests that *MND1* which is an ammonia-oxidizing bacterium involved in nitrogen fixation and ammonia oxidation, and *Bryobacter* improve soil enzymatic activities accelerating biogeochemical cycle of carbon, nitrogen, and phosphorus, and playing an essential role in the remediation of contaminated soils (Wang W. et al., 2022). On the other hand, *Haliangum* and *Gemmatimonas* have been proposed as beneficial soil bacteria that tend to be increased in healthy soils (Gong et al., 2021; Shang and Liu, 2021). Finally, multiple strains from *Chitinophagaceae* have been isolated and reported as PGPR (Madhaiyan et al., 2015; Dahal et al., 2017; Ke et al., 2018; Lee and Whang, 2020). Therefore, these bacterial genera found in all the five rhizospheres of the desert and Antarctic plant species might have synergistic functionalities that help plant with environmental adversities.

In addition, by comparing the core microbiota at species level of each plant, commonalities were identified among the five plant species included in this study. The Antarctic plants (Da and Cq) shared a core microbiota that included the bacterial species *Occallatibacter riparius, Methylibium petroleiphilum, Flavitalea flava, Candidatus Solibacter usitatus,* and *Candidatus Koribacter versatilis*. For desert plants (Cc, Ei, and Ns), the core microbiota includes *Vicinamibacter silvestris*, *Longimicrobium terrae*, *Gemmatirosa kalamazoonesis*, *Candidatus Solibacter usitatus*, and *Aciditerrimonas ferrireducens*. Most species in the core microbial analyses are newly reported, and their functions and roles in microbial communities are not yet well understood (Rawat et al., 2012; Huber et al., 2016; Liu et al., 2019). Notably, these bacteria have not previously been identified in the rhizosphere of plants in the desert or Antarctic environments. Then, our findings provide pioneering insights into these species’ association with plants’ rhizosphere in extreme environments, where they could constitute key community members.

Our results highlight two species shared within the core microbiome of both Antarctic and Desert plant species. Specifically, *Can S. usitatus* was identified in the rhizosphere of the two Antarctic species under study and in Cc samples from the Atacama Desert. This is an acidobacterium that has been studied due to its large genome (>9 Mb), which harbors a large proportion of mobile genetic elements suggesting ancient acquisition of multiple genes that might provide a selective metabolic, defensive, and regulatory advantage in the variable soil environment (Challacombe and Kuske, 2012). Other studies have shown that this species´ membrane composition is responsive to temperature, pH, and O_2_ (Halamka et al., 2023); and that its genome –from an Arctic isolate– contains conserved genes/gene clusters encoding for modules of the carbohydrate-active enzyme (CAZyme) that might allow the breakdown, utilization, and biosynthesis of diverse polysaccharides to resist the fluctuating temperatures and nutrient-deficient conditions (Rawat et al., 2012). On the other hand, *G. kalamazoonesis* was found within the core microbiome of two Desert plant species (Cc and Ei) and in the Antarctic Da. This species belongs to *Gemmatimonadaceae* and was previously highlighted as a key promising group for mitigating N_2_O emissions under climate change since it showed strong resilience that led to significantly reduced N_2_O emissions in urea-treated soils under simulated climate change conditions (Xu X. et al., 2020; Xu R. et al., 2020). *G. kalamazoonesis* was also found as a biomarker associated with carbon/nitrogen metabolism sensitive to different irrigation modes, being more abundant in water-saving irrigation in paddy soils (Wu et al., 2022). Further studies are necessary to elucidate the effects and functional significance of these bacteria in the rhizosphere ecosystem, as they may also be constituents of a plant’s core microbiome that extends beyond harsh environments. Nevertheless, their occurrence in both Antarctic and desert plants’ rhizosphere suggests that these species hold promise as candidates for plant-beneficial bacteria under stress conditions, such as drought. Hence, conducting comprehensive studies focusing on their specific functions and underlying mechanisms is crucial to determine their role in plant survival in harsh conditions.

Based on the 16S rRNA gene taxonomic assignment, the functional analyses predicted chemoheterotrophy, ureolysis, phototrophy, nitrogen respiration, and nitrate reduction as the main represented functions. Notably, some predicted functions matched the known descriptions of the previously discussed genera and species commonalities, including multiple nitrogen and carbon metabolisms. Particularly, both environments showed potential for chemoheterotrophy. Chemoheterotrophic bacteria are predominant in the rhizosphere and obtain their energy from the carbon sources released by the roots (De Albuquerque et al., 2022). Several studies have described the presence of bacteria with chemoheterotrophic characteristics in different samples from the Antarctic territory, associated with the phyla: *Proteobacteria* (*Alpha* and *Gamma*), *Bacteroidetes,* and *Abditibacteriota* (Bowman et al., 1998; Choi et al., 2007; Lee et al., 2007). Additionally, recent studies demonstrate that autotrophic bacteria utilize trace atmospheric gasses (e.g., dihydrogen and carbon monoxide) as an energy source to sustain CO_2_ fixation in Antarctic desert soil, and this mechanism might be present in arid ecosystems (Meier et al., 2021). Although our analysis did not evidence a large representation of chemotrophy, dark hydrogen oxidation was among the most represented common functions in the samples. Then, this type of energy metabolism might offer a minimalist strategy for primary production in extreme desert environments, as previously postulated (Bay et al., 2021). Such a strategy could enhance soil fertility and indirectly facilitate plant colonization in oligotrophic environments.

Furthermore, other functions may play a significant role in withstanding adverse conditions. Recent research supports that using ureolytic bacteria helps mitigate cadmium (Cd) toxicity in Pakchoi plants, as Wei et al. (2022) described. Additionally, the presence of these bacteria promotes the precipitation of calcium carbonate (CaCO_3_), which effectively binds soil particles. This phenomenon has several beneficial implications, including erosion control, bioremediation of heavy metals, and overall soil restoration (Omoregie et al., 2021). Therefore, the ureolysis function, overrepresented in this study, might benefit the plant by improving the soil’s aggregation, leading to positive outcomes such as improved water retention and increased functional activity within the community, particularly in nutrient cycling. In addition, bacteria involved in carbon metabolism, such as chitinolysis and cellulolysis, were increased in soybean rhizosphere under nutrient-deficient conditions, suggesting the need for alternative carbon/nitrogen resources (Chang et al., 2019). Those functions might also be relevant in desertic conditions due to low nutrient availability. The chitinolysis activity has particular importance within biological control; by producing these enzymes, the microorganisms can inhibit the growth of many fungal diseases that seriously threaten crop production, constituting natural biofungicides that could replace chemical fungicides (Swiontek Brzezinska et al., 2014). Moreover, for maize rhizosphere, the relative abundances of functional groups related to chemoheterotrophy, ureolysis, phototrophy, and photoheterotrophy were significantly associated with PGPR inoculation treatments and the fertilization treatments, where the organic matter cycling was improved (Chen et al., 2021). A recent study evidenced that drought may be a more potent driver of climate changes to soil communities than nitrogen or phosphorus deposition (Preece et al., 2020). Given the low water availability in Antarctica and the Atacama Desert, the community structure and function patterns described here offer a promising source to find new PGPR consortia capable of improving plant response to drought.

Remarkably, no significant functional differences were observed for shared taxa (781 sequences) compared to the predictions made using all 3,019 unique sequences. This finding implies that the community structure shared across all five plant species might be the primary determinant of community functioning. Also, given the wide range of taxa associated with plant growth-related functions, the bacterial commonalities described in this study could work as a consortium that collectively maintains key rhizosphere functions and benefit plant survival in poly-extreme environments. However, it is important to acknowledge that our functional predictions rely on 16S rRNA assignments, which should be further validated through metagenomics and meta-transcriptomics analyses. Consequently, it is essential to verify the effects of this bacterial consortium, specifically incorporating species from *Bryobacter, Hallangium*, *Chitinophagacea*, and *Gemmatimonadaceae* taxa, on plant resilience in harsh environmental conditions.

## 5. Conclusion

Our study described the composition and functional prediction of the rhizosphere of Antarctic plants (Da and Cq) and Atacama Desert plants (Cc, Ei, and Ns) based on metabarcoding of complete 16S rRNA gene. Our results suggested that plants may recruit and conserve common bacteria that promote their growth and survival in inhospitable environments of Antarctic and desert habitats. We observed the taxonomic composition of both environments, emphasizing common genera: *Haliangium, Bryobacter*, unclassified (family *Chitinophagaceae* and *Gemmatimonadaceae*), *Bacillus,* and *MND1*, that have been previously related to plant growth promotion. We also reported the microbial core of the rhizosphere of Antarctic and desert plants. For Da and Cq plants, we found the species *Occallatibacter riparius, Methylibium petroleiphilum, Flavitalea flava, Candidatus Solibacter usitatus,* and *Candidatus Koribacter versatilis*. In the case of Cc, Ei, and Ns plants, we reported the species *Vicinamibacter silvestris, Longimicrobium terrae, Gemmatirosa kalamazoonesis, Candidatus Solibacter usitatus,* and *Aciditerrimonas ferrireducens*. We also predicted functions such as ureolysis, nitrogen fixation, and chitinolysis that might be relevant for plant survival under extreme conditions. Consequently, the identified commonalities in genera and species emphasized in this research could hold considerable potential as prospective candidates for the development of biostimulant inocula to enhance plant growth.

## Data availability statement

The data presented in the study are deposited in the SUB13331278 repository, accession number PRJNA972307 (https://www.ncbi.nlm.nih.gov/bioproject/PRJNA972307).

## Author contributions

MJC and KL: conducted experiments, analysed the data and wrote the manuscript. KN-M, OG-E, AS, and PB: bioinformatics analysis, revision, editing, discussion, and proofreading of original draft. BV, GG, MC, AD, CI, and FS: revision and editing of original draft. LBr: revision, editing, discussion and proofreading of original draft. LBa and PZ: supervision, revision, editing, discussion and proofreading of original draft, and Project administration. All authors contributed to the article and approved the submitted version.

## Funding

This study was supported by the Chilean National Agency for Research and Development (ANID) (Grant number FSEQ210003) and Chilean National Fund for Scientific and Technological Development (FONDECYT, Grant number 1210563).

## Conflict of interest

The authors declare that the research was conducted in the absence of any commercial or financial relationships that could be construed as a potential conflict of interest.

## Publisher’s note

All claims expressed in this article are solely those of the authors and do not necessarily represent those of their affiliated organizations, or those of the publisher, the editors and the reviewers. Any product that may be evaluated in this article, or claim that may be made by its manufacturer, is not guaranteed or endorsed by the publisher.

## Footnotes

^1^ https://github.com/rrwick/Porechop

^2^ http://www.loucalab.com/archive/FAPROTAX/
