# Supplementary material for: Commonalities between the Atacama Desert and Antarctica rhizosphere microbial communities
Source: Front Microbiol. 2023 Jul 19;14:1197399. doi: 10.3389/fmicb.2023.1197399 (PMC10395097; doi:10.3389/fmicb.2023.1197399)
Supplement: Supplementary file 1 [file Table_1.DOCX]

Supplementary Material

Commonalities between Atacama Desert and Antarctica rhizosphere microbial communities.

María José Contreras^1†^, Karla Leal^1†^, Pablo Bruna^1^, Kattia Nuñez-Montero^2^, Olman Goméz-Espinoza^3^, Andrés Santos^4^, León Bravo^5^ Bernardita Valenzuela^6^, Francisco Solis^6^, Giovanni Gahona^6^, Mayra Cayo^6^, Alejandro Dinamarca^7^, Claudia Ibacache^7^, Pedro Zamorano^8*^, Leticia Barrientos^2*^.

1. Universidad de La Frontera, Center of Excellence in Translational Medicine, Faculty of Medicine, Av. Alemania 0458, Temuco, Chile.

2. Facultad de Ciencias de la Salud, Instituto de Ciencias Biomédicas, Universidad Autónoma de Chile, Temuco 4810101, Chile.

3. Instituto Tecnológico de Costa Rica, Biotechnology Research Center.

4. Universitat Autònoma de Barcelona, Departament de Genètica i de Microbiologia, Institut Biotecnologia i de Biomedicina, 08193 Cerdanyola del Vallès, Barcelona, Spain.

5. Department of Agricultural Sciences and Natural Resources, Faculty of Agricultural Sciences and Environment, Universidad de La Frontera, Temuco, Chile.

6. Universidad de Antofagasta, Antofagasta Institute.

7. Centro de Micro-Bioinnovación, Universidad de Valparaíso, Valparaíso 2360102, Chile

8. Universidad de Antofagasta, Biomedical Department and Antofagasta Institute.

†These authors contributed equally to this work.

*** Correspondence:** Leticia Barrientos Díaz- [Leticia.barrientos@ufrontera.cl](mailto:Leticia.barrientos@ufrontera.cl)

Pedro Zamorano- [Pedro.zamorano@uamail.cl](mailto:Pedro.zamorano@uamail.cl)

# Supplementary Figures


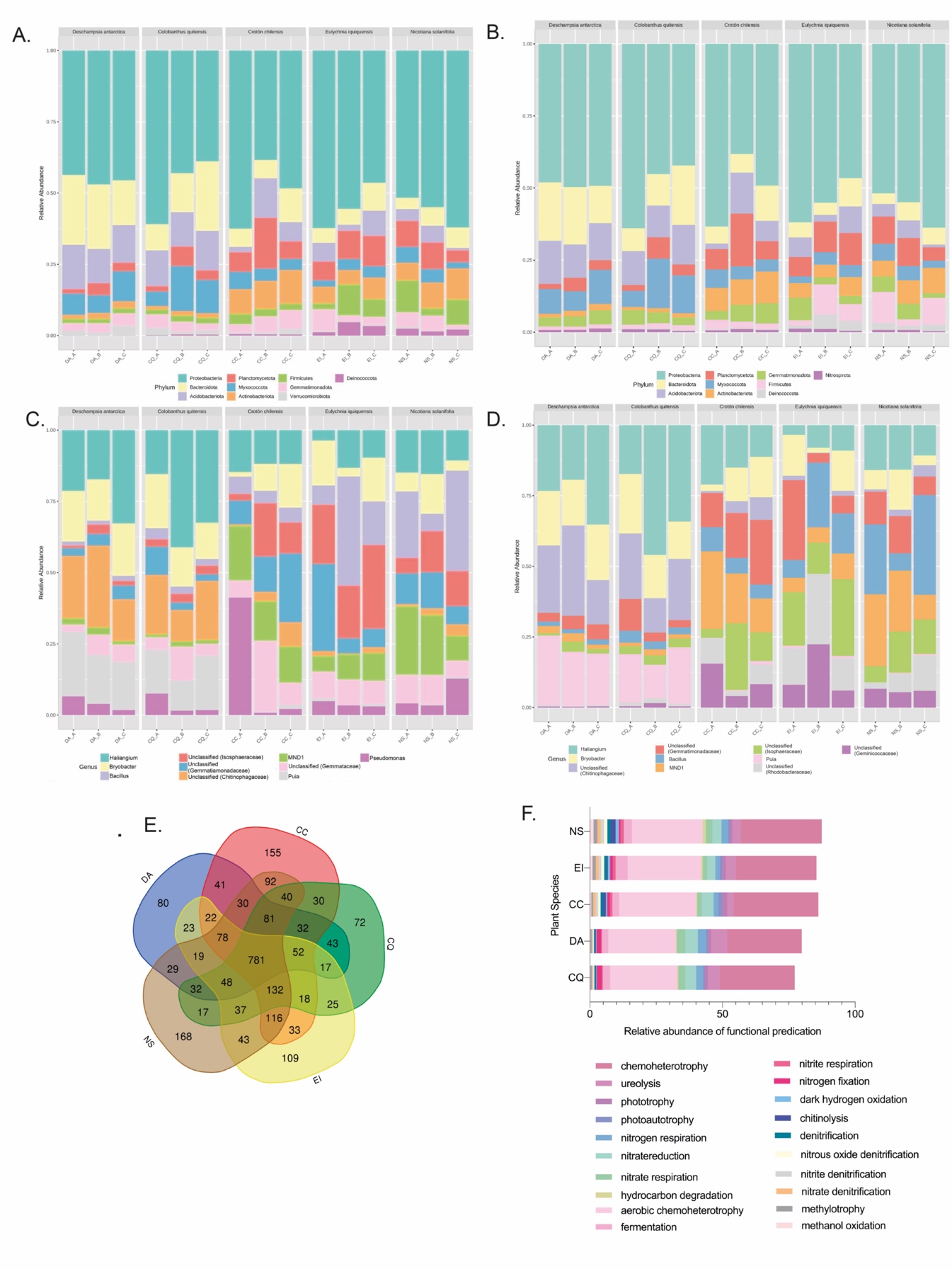


**Supplementary Figure 1.** **Taxonomic composition in the rhizosphere of Antarctic (Da and Cq) and Atacama Desert plants (Cc, Ei and Ns).** **(A)** Mean relative abundances of major phylum-level taxa of total rhizobacterial communities. **(B)** Mean relative abundance of the microbial community composition at the genus of total rhizobacterial communities. **(C)** Mean relative abundances of major phylum-level taxa of core rhizobacterial communities. **(D)** Mean relative abundance of the microbial community composition at the genus of core rhizobacterial communities. **(E)** Venn diagram representing the 16S rRNA sequences shared between the Antarctic and desert rhizosphere bacterial communities. **(F)** Functional prediction of the rhizosphere bacterial communities using the total rhizosphere community (3019 unique sequences). *Deschampsia Antarctica (Da), Colobantus quitensis (Cq), Croton chilensis (Cc), Eulychnia iquiquensis (Ei), Nicotiana solanifolia (Ns).* Letters _A _B _C show biological replicates.


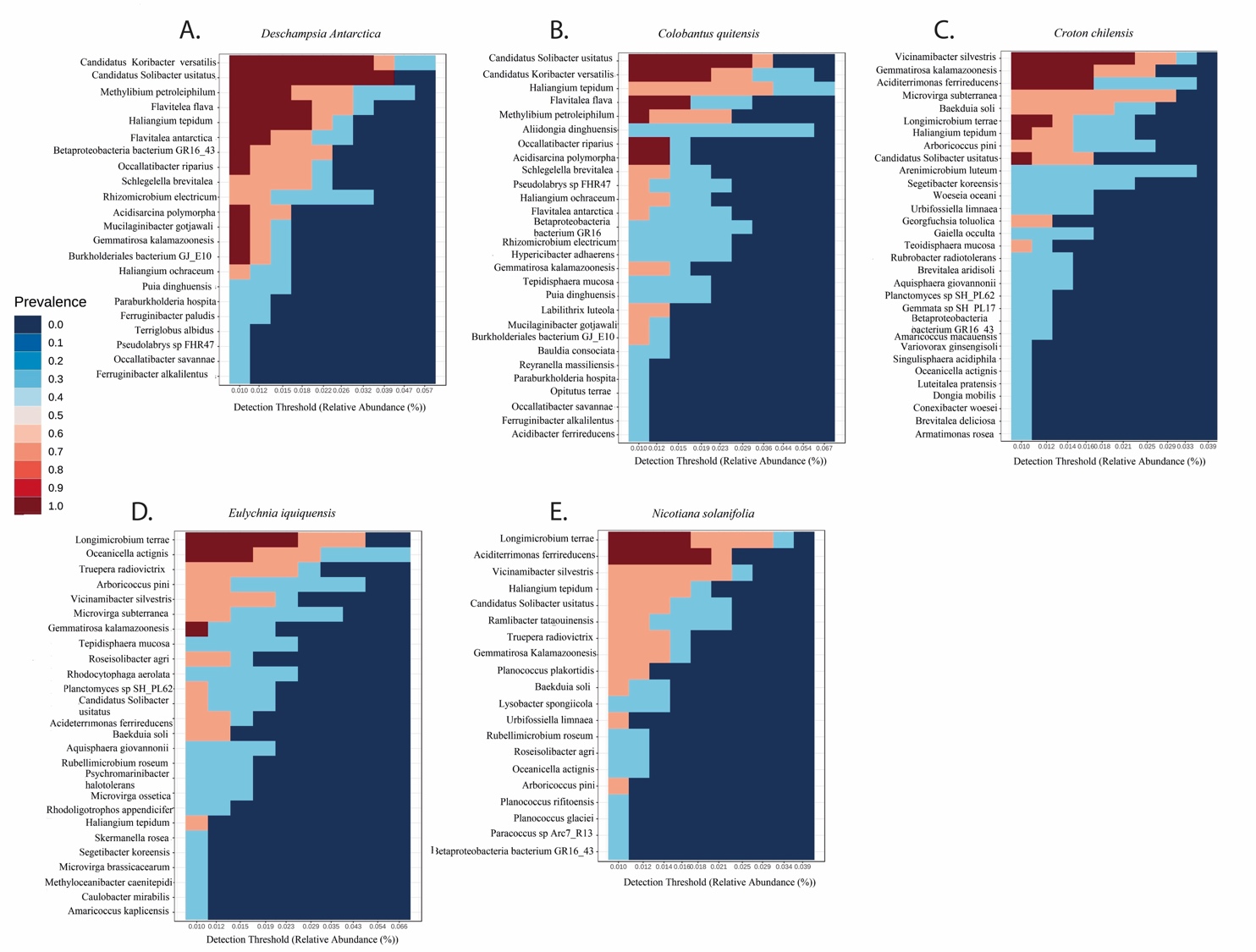


**Supplementary Figure 2.** **Core microbiomes by each plant rhizosphere.** **(A)** *Deschampsia Antarctica;* **(B)** *Colobantus quitensis;* **(C)** *Croton chilensis;* **(D)** *Eulychnia iquiquensis;* **(E)** *Nicotiana solanifolia.* The x-axis indicates the detection threshold in relative abundances.
